# Selective deposition of dietary α-Lipoic acid in mitochondrial fraction and its synergistic effect with α-Tocoperhol acetate on broiler meat oxidative stability

**DOI:** 10.1186/1476-511X-12-52

**Published:** 2013-04-23

**Authors:** Rashida Parveen, Ali Asghar, Faqir M Anjum, Muhammad I Khan, Muhammad Sajid Arshad, Ammara Yasmeen

**Affiliations:** 1 National Institute of Food Science and Technology, University of Agriculture, Faisalabad, Pakistan; 2 Food and Biotechnology Research Center, PCSIR Laboratories complex, Lahore, Pakistan

**Keywords:** α-Lipoic acid, α-Tocopherol acetate, Broiler meat, Oxidative stability, TBARS, Antioxidant activity, Sub-cellular membrane (mitochondria)

## Abstract

The use of bioactive antioxidants in feed of broiler to mitigate reactive oxygen species (ROS) in biological systems is one of promising nutritional strategies. The aim of present study was to alleviate ROS production in mitochondrial fraction (MF) of meat by supplemented dietary antioxidant in feed of broiler. For this purpose, mitochondria specific antioxidant: α-lipoic acid (25 mg, 75 mg and 150 mg) with or without combination of α-tocopherol acetate (200 mg) used in normal and palm olein oxidized oil (4%) supplemented feed. One hundred and eighty one day old broiler birds were randomly divided into six treatments and provided the mentioned feed from third week. Feed intake, feed conversion ratio (FCR) remained statistically same in all groups while body weight decreased in supplemented groups accordingly at the end of study. The broiler meat MF antioxidant potential was significantly improved by feeding supplemented feed estimated as 1,1-di phenyl-2-picrylhydrazyl (DPPH) free radical scavenging activity, 2,2-azinobis-(3- ethylbenzothiazoline-6-sulphonic acid) (ABTS+) and thiobarbituric acid reactive substances (TBARS). The maximum antioxidant activity was depicted in group fed on 150 mg/kg α-lipoic acid (ALA) and 200 mg/kg α-tocopherol acetate (ATA) (T4) in both breast and leg MF. Moreover, TBARS were higher in leg as compared to breast MF. Although, oxidized oil containing feed reduced the growth, lipid stability and antioxidant potential of MF whilst these traits were improved by receiving feed containing ALA and ATA. ALA and ATA showed higher deposition in T4 group while least in group received oxidized oil containing feed (T5). Positive correlation exists between DPPH free radical scavenging activity and the ABTS + reducing activity. In conclusion, ALA and ATA supplementation in feed had positive effect on antioxidant status of MF that consequently diminished the oxidative stress in polyunsaturated fatty acid enriched meat.

## Introduction

Polyunsaturated fatty acids *e.g.* linoleic (C_18:2_n-6) and linoleinic (C_18:3_n-3) acids are now classified as conditionally essential for human and class mammalian because of deficient enzyme which is responsible to synthesize these acids in plants [1]. However, the other unsaturated fatty acids such as ararchidonic acid (C_20:4_n-6), eicosapentaenoic acid (C_20:5_n-3), docosahexaenoic acid (C_22:6_n-3) that are essential components of cellular membranes synthesized from them are very prone to autoxidation [2]. This leads towards high production of potential precursor of very reactive cytotoxic aldehydes in tissues and foods and become the source of oxidation stress in tissues [3,4]. These aldehydes can be major contributing factor in several pathological disorders such as atherosclerosis, inflammation, arthritis, ageing, Alzheimer’s and Parkinson’s diseases [5] The lipids associated with the subcellular organelles are susceptible to oxidation by reactive oxygen species (ROS). These ROS are generated mainly in mitochondria [6] and other subcellular organelles are also affected like microsomes [7] Lysosomes [8] Peroxisomes [9]. Moreover, mitochondria are known to be a rich source for the production of ROS that leads towards lipid peroxidation (6). Previous studies showed defensive mechanism in tissue that can be strengthened by supplementation of feed with vitamin E (in the form of ATA). To reduce or minimized the lipid peroxiation in meat, numerous synthetic and natural antioxidants and antioxidant enriched plant extracts are used in feeds of broiler. One of these antioxidants, α-tocopherol acetate is major lipophilic free radical scavenger *in vivo* for the protection of membranal lipids [7,10]. It has been observed that ATA not only deposit linearly in microsomal membrane to stabilize phospholipids, but also improved the FCR, growth performance [11] whilst TBARS was reduced [12]. α-Lipoic acid is a one of the most active biological antioxidant plays a pivotal role in energy metabolism. It is unique, among antioxidants, owing to its powerful antioxidant properties in both reduced (dihydrolipoic acid) and oxidized (ALA) condition. Dihydrolipoic acid plays an important role in recycling of other radical scavengers such as glutathione, ascorbate and ATA [13-15] protects membrane from oxidation. Exogenous supply of α-lipoic acid appears to impart a variety of significant positive effects in biological system including free radical scavenging potential [16]. It is well documented that feeding ALA lower oxidative damage thus improves mitochondrial stability [17]. Oxidized oil caused a significant reduction in broiler body & carcass weights that undergo rapid oxidative degradation thus had an adverse effect on stability of membranal lipids [18]. Antioxidants *i.e.* ATA and Butylated hydroxyl anisole/Butylated hydroxyl toluene supplementation improved growth and oxidative stability of meat especially in microsome than mitochondria fraction [10]. Nature has equipped us and all other eukaryotes with different defense mechanism to guard against these adversaries, sometimes the defense is not enough in biological systems. Hence the present studies we intend to examine the antioxidative potential of mitochondria of meat that fortified by mitochondrial-specific bio antioxidant, ALA supplementation in the feed of broiler as model biological system. Thus resultant meat will be considered healthier and nutritive for human consumption.

## Material and methods

### Reagents, chemicals and materials

ALA and ATA were purchased from Puritan’s Pride, United States of America and Merck (Merck K Ga A, Darmstadt, Germany), respectively. The reagents used for the present study were 1,1-di phenyl-2-picrylhydrazyl (DPPH), 2,2-azinobis-(3- ethylbenzothiazoline-6-sulfonic acid) ABTS+, hydrogen peroxide, ferrous sulphate, trichloroacetic acid, thiobarbituric acid, hydrochloric acid, ascorbic acid, urea, sodium dodecyl sulfate (SDS), pyrogallol, acetonitrile, methanol, ethanol and other reagents were purchased from Sigma-Aldrich Tokyo, Japan. The oxidized oil was prepared from palm olein oil (purchased from a local store) by heating at 180°C for 8.5 h. The composition of the control feed was 39 g of corn, 6.0 g of wheat, 2.07 g of broken rice, 5.60 g of polished rice, 2.20 g of cottonseed meal, 2.0 g of canola meal, 2.30 g of corn gluten 60%, 12.40 g of sunflower meal, 15.0 g of soybean meal, 6.60 g of fish meal, 3.0 g of soya oil, 0.15 g of L-lysine, 0.08 g of DL-methionine, 1.20 g of dicalcium phosphate, 0.90 g of limestone, 0.50 g of premix, and 4.0 g of molasses (total 100 g). The metabolized energy feed was 2900 kcal/kg, crude protein (21.03%), and fiber (6.50%). After acclimation, the experimental birds were fed for 21 days with the standard diet-containing placebo (Group 1, control).

### Experimental birds

Experimental birds were reared at Poultry Research Center, University of Agriculture Faisalabad (UAF), Pakistan and analysis of meat was performed at National Institute of Food Science and Technology, UAF. One hundred and eighty, one day old, broiler birds of Hubbard strain were purchased from Jadid Hatchery Faisalabad. The birds were weighed individually and randomly divided in to six experimental units with three replicates (10 birds in each replicate). The birds in each experimental unit were kept in separate disinfected pens (4 ft × 3 ft × 1.5 ft). Feed along with fresh and clean water was given *ad-libitum*. From third week, feed was provided to broiler that supplemented with ALA and ATA as indicated in Table 1. Percent change in body weight (BW), feed consumption (FC) and feed conversion ratio (FCR) was recorded at the termination of study. At the end of 6 weeks, three birds from each replicate were randomly selected and slaughtered according to Islamic Ethical Guidelines. Breast and leg meat were separated and packed in polyporpylene zip lock bag and stored in freezer at −80°C (Sanyo, Japan).

**Table 1:** Treatments plan of feed supplemented with α-lipoic acid and α-tocopherol acetate

**Treatment**	**Description of feed**
**T**_**1**_	Control
**T**_**2**_	α-Lipoic acid (25 mg) + α-tocopherol acetate (200 mg)/ Kg of feed
**T**_**3**_	α-Lipoic acid (75 mg) + α-tocopherol acetate (200 mg)/ Kg of feed
**T**_**4**_	α-Lipoic acid (150 mg) + α-tocopherol acetate (200 mg)/ Kg of feed
**T**_**5**_	Oxidized oil (4%)/ Kg of feed
**T**_**6**_	Oxidized oil (4%) + α-Lipoic acid (150 mg) + α-tocopherol acetate (200 mg)/ Kg of feed

### Isolation of mitochondrial fractions of meat

Mitochondrial fraction of meat was isolated by following the method of [19] and briefly described herein.

#### Homogenization

Breast and leg meat sample (6 ± 0.01 g) was homogenized by using 20 mL of 0.1 M phosphate buffer containing ethylene diamine tetra acetic acid (EDTA) at 7.4 pH in 50 mL polypropylene tube for 10 min at 4000 *g* in ice containing backer, 15 sec rest was given after 60 sec. Connective tissues were removed from meat homogenate through filtration by using muslin cloth.

#### Centrifugation

The filtrate was centrifuged at 1,000 × *g* for ten min at 4°C to remove the nuclear fraction from meat filter homogenate. Supernatant (40 mL) was separated and centrifuged to sediment mitochondria at 10,000 *× g*. Mitochondria was collected and stored at −80°C for further analysis. Mitochondrial fraction was subjected to estimate the protein content by follow the Lowry method [20] for equalization of mitochondrial fraction (MF) sample on the basis of protein content of breast and leg meat. Stock solution of standard was prepared 1 mg/ml of bovine albumin serum for protein quantification.

### Antioxidant potential of mitochondrial fraction of breast and leg meat

Antioxidant potential of the mitochondrial fraction of breast and leg meat was measured by DPPH free radical scavenging activity, ABTS + reducing activity and TBARS methods.

### DPPH free radical scavenging activity

The antioxidant activity of the mitochondrial fractions was estimated by measuring their scavenging abilities to DPPH stable radicals. The DPPH assay was performed as described by [21]. Breast and leg meat sample 125 μL mixed with 0.0012 M DPPH solution followed by the addition of 95% MeOH up to final volume of 4 mL. The absorbance of the resulting solution and the blank was recorded estimated after 30 min at room temperature. The disappearance of DPPH was read spectrophotometrically (U-2001, model 121-0032 Hitachi, Japan) at 515 nm. Inhibition of free radicals by DPPH in percent (%) was calculated in following way.

$$\mathrm{Inhibition}\;\left(\%\right)=\left[100\times \left(A\;\mathrm{blank}–A\;\mathrm{sample}\right)÷A\;\mathrm{blank}\right]$$

### ABTS + reducing activity

ABTS + reducing activity was measured as the method described by [22]. ABTS + (7 mM) solution was prepared in distilled water. Radical cation of ABTS was produced by reacting ABTS stock solution with 2.45 mM potassium persulfate (final concentration) in dark for 12 h at room temperature to allow the completion of radical generation. This solution was diluted with EtOH and adjusted the absorbance at 734 nm as 0.70 ± 0.03. The diluted ABTS + solution (6 mL) were added to 40 μL mitochondrial sample and absorbance was measured by spectrophotometer (U-2001, model 121-0032 Hitachi, Japan) at 734 nm using ethanol as blank. The percentage of inhibition was estimated by following formula.

$$\begin{array}{l} \mathrm{ABTS}+\mathrm{reducing}\;\mathrm{activity}\;\left(\%\right)\\ \;=\left[\left(A\;\mathrm{control}–A\;\mathrm{sample}\right)÷A\;\mathrm{control}\right]\times 100 \end{array}$$

### Lipid peroxidation stability determine by thiobarbituric acid reactive substances (TBARS)

The peroxidation stability of mitochondria was determined by TBARS assay [21]. The peroxidative reaction was initiated by adding ferrous sulphate and hydrogen peroxide to the membrane suspension held in a water bath at 37°C. One milliliter sample prepared in buffer solution (0.1 M KCl; 0.05 M NaOH; 0.13 M lactic acid) with pH 5.3–5.4 was withdrawn at 30 min intervals for a period of 120 min and added to a same volume of solution of thiobarbituric acid (0.4%) trichloroacetic acid (10%) and hydrochloroic acid (0.25 N). The mixture was heated in a boiling water bath for 15 min and then cooled. After centrifugation, the absorbance of the supernatant was determined at 532 nm. The extent of membrane lipid peroxidation was calculated by using the formula.

$$\begin{array}{l} n-\mathrm{Moles}\;\mathrm{of}\;\mathrm{malondialdehydes}\\ \;=[\left\{\left(\mathrm{Abs}\;\mathrm{sample}–\mathrm{Abs}\;\mathrm{blank}\right)\times \mathrm{Total}\;\mathrm{sample}\;\mathrm{volume}\right\}\\ \;\left\{0.00156\times 1000\;\left(\mathrm{per}\;\mathrm{mL}\right)\right\}] \end{array}$$

### Quantification of α-tocopherol acetate from mitochondrial fraction of breast and leg meat by HPLC isocratic system

Alpha-tocopherol acetate was estimated by HPLC according to method described by [7] with slightly modifications. Mitochondrial sample (500 mg) was mixed with 5% ascorbic acid (0.5 mL) prepared in nitrogen saturated water followed by 6 M urea (1 mL). Solution was flushed with nitrogen gas and vortexed for 1–2 min to dissolve the sample. One mL of 0.1 M sodium dodecyl sulphate (SDS) solution was added and vortex again followed by the addition of 4 mL ethanol (95%) contain 1% pyrogallol. Sample was vortexed for 30 sec for de-proteination and freeing of ATA from membrane of the cell. After that, petroleum ether (8–10 mL) was added and vortexed for 2 min followed by centrifuged at 5000 × *g* for 4–5 min. Transferred upper solvent layer to a vial or glass and evaporated under nitrogen stream. The pooled solvent layer was added 200 to 500 μl ethyl alcohol flush to facilitate the solubilization of ATA. Samples were filter through microfilter (anspec 0.45 μm) and stored in the dark until further analysis. After the addition of 500 μL methanol, the solution was heated for 1 min at 45°C to dissolve the ATA. The solution was centrifuged at 2,000 × g for 5 min and then the liquid layer was filtered. The filtrate was analyzed for ATA using a Shimadzu HPLC (Kyoto, Japan) equipped with 8 cm × 4.6 mm × 5 μm Shim-Pack CLC (C18) column (Shinwa Chemicals, Kyoto, Japan) at 290 wavelength UV-detectors and one mL flow rate was used and sample (20 μl) was injected. Mobile phase was methanol (100%) used to quantify ATA by using a UV-isocratic chromatographic system.

### Quantification of α-lipoic acid from mitochondrial fraction of breast and leg meat by HPLC gradient system

α-Lipoic acid content was measured from mitochondrial fraction of breast and leg meat by HPLC gradient system [21] with slightly modifications. On protein basis, mitochondrial sample were taken for each treatment in the glass tubes and mixed with 3 mL hexane containing 250 μL isopropanol and vortexed for 30 min. Sample was centrifuged at 1500 × *g* and upper hexane layer was collected in glass tube. This step was repeated twice and pooled the resultant n-hexane layer. For derivatization of ALA, 0.2 mL (1 mg protein/mL) sample was mixed with methanolic sulphric acid (2 mL) and shaken well. The resultant adduct was heat at 80°C for one hour in water bath and vortexed 15 min. Two milliliter of distilled water added to stop the reaction and ALA was separated by petroleum ether (1 mL) thrice and then evaporated the ether content under nitrogen stream and stored. α-Lipoic acid was estimated by injecting 20 μL sample in Shimadzu HPLC (Kyoto, Japan) equipped with a 15 cm × 4.6 mm × 5 μm Shim-Pack CLC (C18) column (Shinwa Chemicals, Kyoto, Japan). The fluorescence detector was operated at excitation and emission wave lengths of 343 and 423 nm, respectively. The mixture of acetonitrile/water (80:20 v/v) used as a mobile phase with flow rate 1.0 mL/min.

### Statistical analysis

Data obtained from various parameters were subjected to one way analysis of variance (ANOVA). Mean and standard deviation of the mean was measured by Statistic 8.1 Program (Analytical Software). Difference among the treatments were calculated by the least-squared difference with a significance level (p < 0.05).

### Ethical approval

The animals were slaughtered according to the Halal ethical guidelines and the approval is given by the head of the National Institute of Food Science and Technology, University of Agriculture, Faisalabad, Pakistan.

## Results and discussion

### Body weight

**Figure 1:**
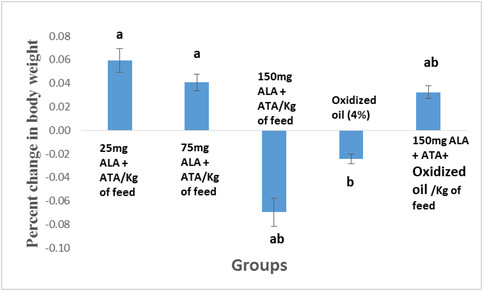
Percent change in body weight as compared to control.

Percent changed in body weight with respect to control was depicted in Figure 1. Growth rate of broiler birds were affected by ALA and ATA supplemented feed. Maximum weight gain was recorded in group receiving lowest level of ALA (25 mg/kg feed) while minimum weight gain was observed in group that fed on 150 mg/kg ALA with 200 mg/kg ATA feed. Oxidized oil showed diminishing effect on growth that significantly decreased as compared to control. However, hindering effect of oxidized oil on growth was reported by the addition of antioxidants in the feed. FC and FCR differed non-significantly throughout the experiment (Table 2). The feed consumption increased with the increase in time period. The feed consumption presented in Table 2 was for the whole experiment (6 weeks) Maximum feed consumption was recorded in 150 mg ALA and 200 ATA/kg (T4) and 150 mg ALA and 200 ATA/kg with 4% oxidized oil containing fee (T6) as 6.19 kg revealed that groups receiving higher dose of α-lipoic acid showed higher feed consumption. Better feed conversion ratio was found in the T2 group containing minimum dose of ALA (25 mg). Previously, it is reported that antioxidant supplementation in feed significantly improved the growth of broilers by consuming higher feed (p < 0.05) [21]. On the other hand, dietary α-lipoic acid had no effect on growth rates [23] The present study showed that the higher concentration of ALA (150 mg) in the feed (T4) suppressed the growth performance of broilers, suggesting that there is an optimum level of ALA supplementation. It is also reported that dietary ALA supplementation reduced food consumption as a response to the inhibition of AMP-activated protein kinase activity in the hypothalamus [24]. Supplementation of oxidized oil (T5) suppressed the growth rate slightly. However, supplementation of ALA and ATA (T6) improved the growth significantly. The results obtained in the present study were consistent with previously reported results [25]. Similar findings were reported that ALA supplementation to a control diet decrease in body weight gain [26].

**Table 2:** Feed consumption (kg) and Feed Conversion ratio (FCR) of broilers used in the study

**Treatments**	**Feed consumption (kg)**	**FCR**
**T**_**1**_	6.17 ± 0.30	1.98 ± 0.099
**T**_**2**_	5.98 ± 0.27	1.83 ± 0.098
**T**_**3**_	6.08 ± 0.32	1.97 ± 0.101
**T**_**4**_	6.19 ± 0.37	2.01 ± 0.095
**T**_**5**_	5.98 ± 0.29	1.93 ± 0.094
**T**_**6**_	6.19 ± 0.34	1.93 ± 0.096

Results are presented as Mean ± SD of three independent measurement.

### Antioxidant potential of mitochondrial fraction of leg and breast broiler meat

Analyses were performed for measuring the antioxidant potential of the mitochondrial fraction of the broiler meat (Table 3). Two types of antioxidant tests were performed to validate the results such as DPPH free radical scavenging activity and ABTS + reducing activity that measured the antioxidants potential of the MF of broiler meat. DPPH is unstable free radical and when mixed with an antioxidant, it donates a hydrogen atom to form a stable DPPH-H molecule. DPPH free radical scavenging activity determines the percent inhibition, when percent inhibition is more it means that there will be more antioxidant activity. The percent inhibition was found maximum in group receiving highest dose of ALA (150 mg/Kg) along with ATA (200 mg/Kg) T4, both in the breast and the leg meat. However, the minimum percent inhibition found in oxidized oil containing feed (T5) both in the breast and leg meat as depicted in the Table 4. The T4 showed significant difference with that of control and also there was significant variations with T2 where the minimum ALA (25 mg) was used in feed. The ABTS+, which is stable free radical cation applicable to both lipophilic and hydrophilic antioxidants, has been used to measure total antioxidative activity [27] breast and leg meat of broiler mitochondrial membrane fed on maximum dose of antioxidants (150 mg ALA and 200 mg ATA) T4 had significantly higher ABTS + reducing activity as compared with control. Moreover, ABTS + reducing activity was found to be the minimum in T5 where the oxidized oil was used. ABTS + reducing activity also significantly higher in T4 compared with T6 where the maximum dose of antioxidants was used with the oxidized oil. Recently [28] published the results that, the breast meat of broiler fed on Gallic acid (0.5 and 1.0%) had significantly higher ABTS + reducing activity than that of control during storage period (7 days) except zero day.

**Table 3:** DPPH free radical scavenging activity and ABTS + reducing activity of mitochondrial fraction of broiler meat

**Treatments**	**DPPH free radical scavenging activity (%)**		**ABTS + reducing activity (%)**	
	**Breast meat**	**Leg meat**	**Breast meat**	**Leg meat**
**T**_**1**_	67.01 ± 3.56^b^	66.79 ± 3.15^b^	29.12 ± 1.41^c^	30.65 ± 1.39^d^
**T**_**2**_	68.18 ± 3.98^b^	67.01 ± 3.37^b^	32.27 ± 1.56^b^	31.89 ± 1.50^c^
**T**_**3**_	70.09 ± 4.01^ab^	69.81 ± 3.77^ab^	33.98 ± 1.63^b^	34.04 ± 1.56^b^
**T**_**4**_	73.51 ± 4.25^a^	72.99 ± 4.03^a^	36.01 ± 1.66^a^	37.56 ± 1.69^a^
**T**_**5**_	61.98 ± 3.79^d^	61.81 ± 3.65^d^	27.91 ± 1.39^d^	28.18 ± 1.23^e^
**T**_**6**_	64.01 ± 3.67 ^c^	64.10 ± 3.36^c^	29.98 ± 1.33^c^	30.05 ± 1.37^d^

Results are presented as Mean ± SD (n = 3), whereas, results in the same column with no superscripts in common differ significantly at P < 0.05.

**Table 4:** α-Lipoic acid contents and α-Tocopherol acetate in breast and leg mitochondrial fraction of broiler meat

**Treatments**	**Alpha-lipoic acid content**		**Alpha-tocopherol acetate content**	
	**Breast meat**	**Leg meat**	**Breast meat**	**Leg meat**
**T**_**1**_	13.69 ± 0.66^d^	14.56 ± 0.69^d^	11.01 ± 0.62^d^	12.31 ± 0.67^d^
**T**_**2**_	15.99 ± 0.69^d^	17.23 ± 0.77^c^	14.98 ± 0.68^c^	16.42 ± 0.78^c^
**T**_**3**_	21.31 ± 0.75^b^	22.01 ± 0.81^b^	20.86 ± 0.72^b^	22.67 ± 0.86^b^
**T**_**4**_	27.41 ± 1.13 ^a^	30.06 ± 1.25^a^	26.04 ± 1.09^a^	28.95 ± 1.27^a^
**T**_**5**_	10.88 ± 0.53^e^	11.54 ± 0.60^e^	9.87 ± 0.54^d^	10.75 ± 0.56^d^
**T**_**6**_	17.59 ± 0.76 ^c^	18.34 ± 0.79^c^	15.93 ± 0.69^c^	17.54 ± 0.78^c^

Results are presented as Mean ± SD (n = 3), whereas, results in the same column with no superscripts in common differ significantly at P < 0.05.

### Lipid peroxidation stability of mitochondrial fraction measured by TBARS

**Figure 2:**
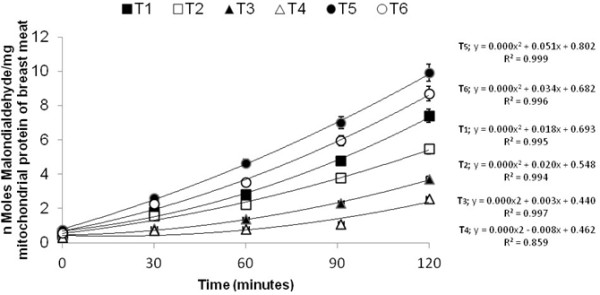
Ferrous sulfate/hydrogen peroxide-initiated auto-oxidation in breast mitochondrial fraction of broiler meat.

**Figure 3:**
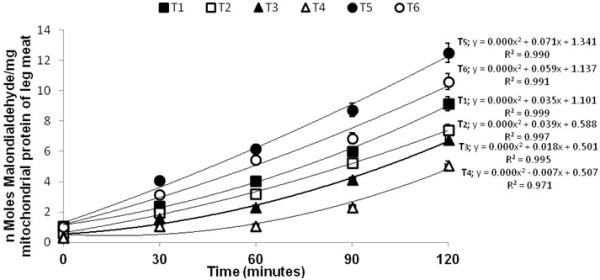
Ferrous sulfate/hydrogen peroxide-initiated auto-oxidation in leg mitochondrial fraction of broiler meat.

Malondialdehyde (MDA) formed from the breakdown of polyunsaturated fatty acids, serves as a convenient index for determining the extent of peroxidation reaction. Malondialdehyde has been identified as the product of lipid peroxidation that reacts with thiobarbituric acid to give a red pigment end products having maximum absorbing capacity at 535 nm. The T4 group (150 mg ALA + 200 mg ATA/kg feed), exhibited less amount of MDA as compare with T1 (control) and oxidized oil showed highest MDA formation in broiler leg mitochondria. The treatments with supplemented antioxidants (ALA and ATA) depicted less production of peroxidation in the form of MDA that is evident from Figures 2 and 3. The trend of auto-oxidation is best explained by polynomial curve. The calculation of MDA is based on protein present in the mitochondria. The protein in mitochondria was calculated by using the linear equation (y = 0.0019 × + 0.0035; R2 279 =0.997). Our findings agreed with [11,29] and [30] that ATA inhibited the formation of MDA. Breast mitochondria fraction also exhibited the same pattern as in the leg mitochondrial fraction of meat. The highest amount of supplemented antioxidant showed more lipid stability thus proved that resultant meat has less autoxidation [31]. reported that α-lipoic acid is an antioxidant and it prevents the lipid peroxidation in rats. Oxidized oil showed highest amount of MDA production in breast as well as in the leg mitochondria as compared with control and leg meat had a higher MDA production because accumulation of linolenic acid as compared with breast meat [32]. The minimum rate of oxidation was observed in group fed with maximum dose of ALA. The rate of oxidation of all group containing small doses of ALA were also lower than control. This suggests that oxidized oil in the diet became a source of free radicals which could destabilize the lipid in sub cellular membrane. Moreover, results of these groups also showed higher rate of oxidation in polyunsaturated fatty acids of leg as compared to breast meat lipids. Correlation between amount of ALA in the feed and the amount of MDA formed in antioxidant enriched broiler leg meat was observed: higher the level of α-lipoic acid in feed of broiler leads to produce less MDA in meat mitochondria. Previously, study reported that oxidative stability of broiler meat was improved by the supplementation of antioxidants such as ATA [29].

### α-Tocopherol acetate (ATA) and α-lipoic acid (ALA) contents in leg and breast mitochondrial fraction of broiler meat

Earlier, it is well documented that antioxidant are more absorbed in microsomes as compared to mitochondrial fraction of meat [7]. The present study is conducted to evaluate the effect of mitochondrial-specific antioxidant *i.e.* α-lipoic acid on mitochondria stability that is very prone to auto-oxidation due to higher accumulation of polyunsaturated fatty acids in its membranes. ATA content increased as the level of ALA increased in the feed of broilers which is evident from Table 4. ATA contents present in the control which showed that it is also naturally present in the meat tissues. The result showed that maximum ALA deposited in T4 and of mitochondrial membrane of breast and leg meat, respectively (Table 4). Control and oxidized treatments also deposited α-lipoic acid but the amount is significant less. Likewise, the accumulation trend of ALA was same as in breast mitochondria meat. It also deposits in control and oxidized oil treatment but deposition rate was progressively elevated in groups that were fed with higher level of ALA. It is suggested from present study results that mitochondrial-specific antioxidants can deposit in mitochondria of the cell and minimized the rate of lipid oxidation and ultimately enhance the quality of meat and meat products. α-Tocopherol acetate concentration was higher in T4 followed by T3, T2 as compared to control. Similarly, ATA contents were higher in T4 of both leg and breast mitochondrial fraction supplemented with ALA (200 mg/kg feed) with constant level of ATA. Gradually the ALA concentration increases, the ATA deposition also increases which is evident from Table 4[13,33]. reported that ALA has synergistic effect with α-tocopherol acetate. But in the leg mitochondria the deposition was higher as compared to breast. Synergistic effect of α-lipoic acid and α-tocopherol acetate was also higher in all supplemented feed treatments of broiler. These results also indicate that two antioxidants have positive effect on the tissues especially mitochondria of meat. Oxidized oil in the feed of broiler was inhibited the deposition of ATA and ALA in meat mitochondrial fraction of meat. In the treatments T2, T3 and T4 the deposition rate of ALA were in increasing pattern as the progressively increased the ALA in the feed (Table 4). Similar trend was observed in the microsomes fractions of the breast and leg meat that were published in elsewhere [7]. The ATA is a biological antioxidant which protects the membranes from oxidation. The supplementation of ATA in feed of animal increase its deposition as in tissues [25,29] reported that free radical production and lipid per-oxidation were significantly decreased/ minimized in muscle as well as has protective effect on lipid peroxidation in mitochondrial fraction animal fed on ATA supplemented diet [34,35]. Moreover, results of ALA obtained in the present study, which are consistent with a previous report, suggest that the ALA has a positive influence on the ATA deposition in meat cell membranes because both synergistically quench ROS, subsequently inhibit oxidative damage in biological systems [36].

## Conclusions

The ALA and ATA significantly enhanced the antioxidant potential of mitochondrial fraction of broiler meat. It was concluded from the present research that supplementation of ALA with ATA improved the lipid stability and mitigate the ROS in mitochondria of the meat tissues thus meat quality of broiler meat is improved. Further, studies are designed to evaluate the storage stability of antioxidants enriched meat, that will consequently helpful for meat industry as well as consumers.

## Competing interests

The authors report no conflicts of interest. The authors alone are responsible for the content and writing of the paper.

## Authors’ contributions

The contribution of the each author for this paper was as follows, RP, MSA, AY carried out the trial of the broiler birds and also collected all the data of the trial. They also arranged all the data and drafted the manuscript. AA and FMA were acting as principal and co-principal investigator of the project and provides technical assistance during research of the broiler and also guided in the analysis and statistical design of research trial. MIK also helped to carry out the analytical research work and analysis of the mitochondrial fraction of meat. It is also confirmed that all the authors read and approved the final manuscript.
